# A Case of Euthyroid Steroid-Responsive Encephalopathy With Subacute Dementia

**DOI:** 10.7759/cureus.17689

**Published:** 2021-09-03

**Authors:** Rebecca John, Abhigyan Datta, Sujith Ovallath

**Affiliations:** 1 Department of Neurology, Kannur Medical College, Anjarakandy, Kannur, IND; 2 Department of Medicine, Maulana Azad Medical College, New Delhi, IND

**Keywords:** hashimoto’s encephalopathy, hashimoto’s thyroiditis, euthyroid, basal ganglia deposition, subacute dementia, cognitive assessment, sreat

## Abstract

Steroid-responsive encephalopathy associated with autoimmune thyroiditis (SREAT), frequently termed as Hashimoto’s encephalopathy (HE), is characterized by reversible encephalopathy with the presence of elevated antithyroid antibodies. The condition was initially described due to its association with Hashimoto’s thyroiditis. We report a case of euthyroid HE presenting as subacute dementia. A 50-year-old woman presented with progressive memory decline for six weeks. Thyroid function tests, thyroid ultrasound, and cerebrospinal fluid analysis were unremarkable. Electroencephalogram showed generalized slowing with triphasic waves. On magnetic resonance imaging, T1 weighted images revealed hyperintensity in bilateral basal ganglia. Antithyroglobulin and antithyroid peroxidase were markedly elevated. She improved remarkably on tablet prednisolone 60 mg once daily, confirming the suspicion of steroid-responsive encephalopathy. Thus, we conclude that patients with subacute cognitive decline could be screened for antithyroid antibodies in the dementia workup despite their euthyroid status.

## Introduction

Hashimoto’s encephalopathy (HE) is a rare cause of reversible cognitive decline [1]. The first case was reported in 1966 by Brain et al. with symptoms of hallucinations, altered mental status, tremor, and elevated thyroid antibodies [antithyroglobulin (anti-TG) antibodies-normal < 4 IU/mL and antithyroid peroxidase (anti-TPO) antibodies-normal < 4 IU/mL] [2]. The hypothesized mechanism includes inflammation of the brain, disruption of cerebral microvasculature secondary to autoantibody or immune complex deposition, and rarely vasculitis [3-6]. Here, we present a case of steroid-responsive encephalopathy with no overt evidence of thyroiditis from South India.

## Case presentation

A 50-year-old right-handed woman with a history of diabetes and hypertension was brought into the clinic by her husband with progressive memory loss for the past six weeks. Initially, she had amnesia of recent daily activities like bathing, having meals, and difficulty in finding words. This further progressed within days and thereafter had difficulty identifying the bathroom, wandering around the house, and often forgetting to turn off the stove after cooking. Her husband had also noticed excessive daytime sleepiness and lethargy. There was no history of loss of consciousness, convulsions, psychosis, and weakness.

On examination, vital signs, cardiovascular exam, and pulmonary exam were unremarkable. Results of the neurological examination noted altered mental status with a Glasgow Coma Scale of 14. Higher mental functions revealed defective recall of immediate and recent events, however, remote memory was intact. Montreal Cognitive Assessment revealed a score of 12. No cranial nerves, focal motor, or sensory deficits were noted. At this point, a provisional diagnosis of subacute dementia was considered and required further evaluation to rule out the treatable causes of acquired cognitive decline.

A complete blood count showed hemoglobin 12.1 g/dL, white blood cell count 10.9×10^9^/L, and platelets 31×10^9^/L. A complete metabolic panel showed random blood glucose 110 mg/dL, creatinine 1.1 mg/dL, blood urea nitrogen 16 mg/dL, alanine aminotransferase 26 U/L, and aspartate aminotransferase 26 U/L. Other laboratory tests showed erythrocyte sedimentation rate 18 mm/h, C-reactive protein 15.9 mg/L, serum ammonia was 12.1 umol/L, serum ceruloplasmin 15.7 mg/d, and antinuclear antibody (ANA) was negative. A lipid panel revealed total cholesterol 101 mg/dL, triglycerides 200 mg/dL, low-density lipoprotein 41 mg/dL, and high-density lipoprotein 18 mg/dL. The HIV and venereal disease research laboratory test (VDRL) panel was negative. Thyroid function studies were normal, revealing T3-9.93 ng/mL, T4-5.74 ug/dL, thyroid-stimulating hormone (TSH)-2.27 IU/mL, and no reduced echogenicity on thyroid ultrasound.

**Figure 1:**
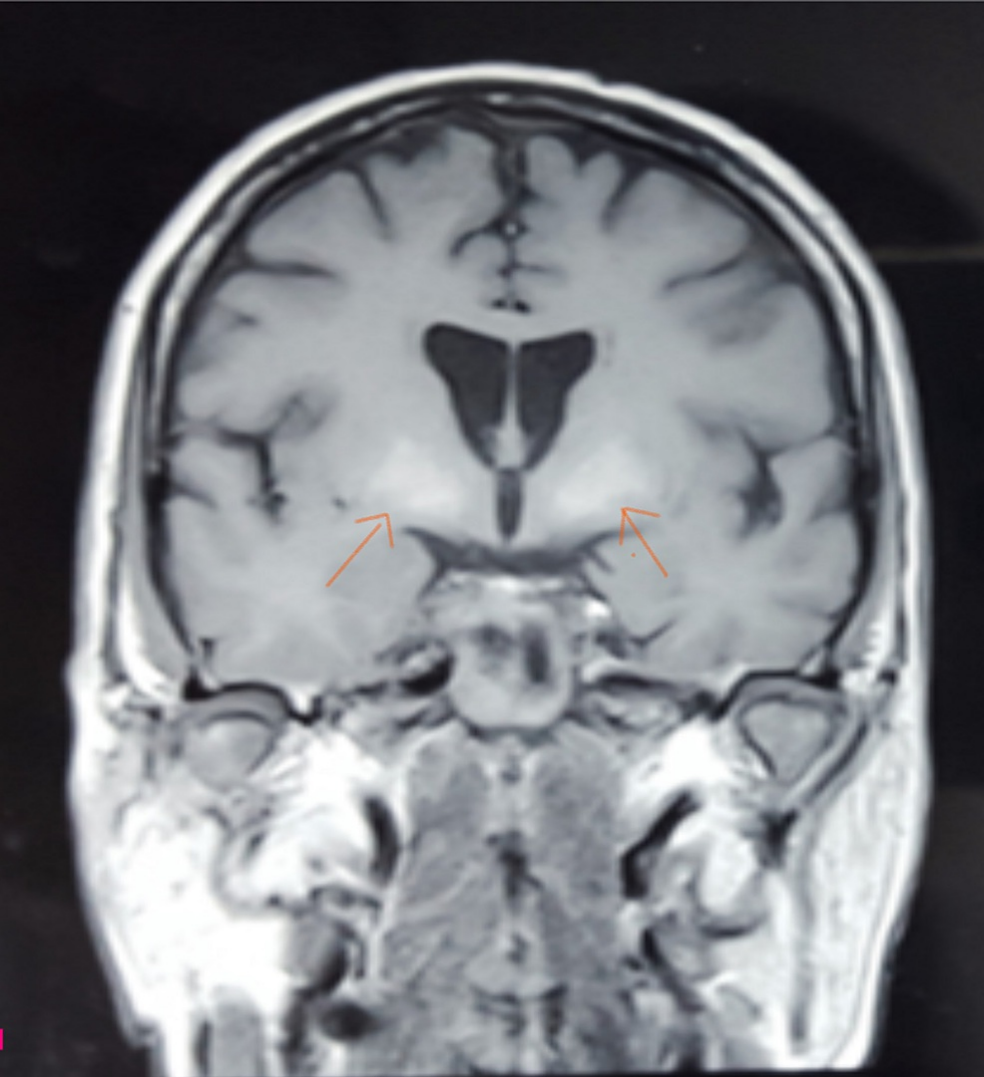
Magnetic resonance imaging coronal view of the brain showing T1 weighted image revealing hyperintensity in bilateral basal ganglia due to mineral deposition. Both the arrows point out to the hypertense foci at the basal ganglia bilaterally on a T1-weighted MRI image that suggests mineral deposition.

Lumbar puncture and cerebrospinal fluid (CSF) study were unremarkable with normal protein levels. Electroencephalogram (EEG) showed generalized slowing and occasional triphasic waves. Magnetic resonance imaging (MRI) revealed T1 hyperintensity in bilateral basal ganglia possibly due to secondary mineral deposition, and small vessel ischemic changes were not visualized (Figure 1).

Antithyroid peroxidase antibody was above 1300 IU/mL, and antithyroglobulin antibody was more than 500 IU/mL. A suspicion of Hashimoto’s encephalopathy was made despite the normal thyroid panel. The patient was started on tablet prednisolone 60 mg once daily with monitoring of blood glucose level. She demonstrated a robust improvement in cognitive status within one week. Montreal Cognitive Assessment revealed a score of 20. One month after discharge, she reported further improvement in memory and cognition, with a complete return to normal activity and no symptoms of recurrence after tapering the dosage of steroids. She has been on a maintenance dose of oral prednisolone 10 mg once daily, and no relapse was noted on her yearly follow-up. Currently, she is stable with long-term steroid management. We plan to add immunosuppressants if she relapses or significant adverse effects of prolonged steroids poses a risk.

## Discussion

Hashimoto’s encephalopathy (HE) is an uncommon disorder with a prevalence of 2.1/100,000 among the general population [4]. This entity has been quoted repeatedly in the literature by the scientific community and illustrates the need for a detailed understanding of pathophysiology. 

HE is a neuropsychiatric manifestation that may present acutely as cerebral ischemia, psychosis, and seizure, or as a subacute/chronic progression of cognitive decline, delirium, depression, myoclonus, and tremors [6-8]. It is predominantly seen in women with elevated antithyroid antibodies and is considered to be a diagnosis of exclusion [7]. Our patient presented similarly with subacute dementia and confusion. We first evaluated possible treatable etiologies of acquired cognitive decline, such as syphilis, HIV, cerebral vasculitis, and B12 deficiency. 

After excluding the above causes with laboratory investigations and imaging, we evaluated HE as the possible etiology, the diagnosis of which traditionally requires the presence of encephalopathy with neurologic manifestations, the presence of increased antithyroid antibodies, exclusion of infectious and metabolic disorders, and rapid clinical improvement after immunomodulation [9]. In our patient, the diagnosis was confirmed by the elevated antithyroid antibodies and rapid response to oral prednisolone within one week of treatment, with remission on follow-up.

Due to the presence of elevated antithyroid antibodies, one might correlate a history of Hashimoto’s thyroiditis leading to HE. The criteria for diagnosing Hashimoto's thyroiditis require a combination of clinical features, elevated thyroid antibodies, and a thyroid sonogram showing reduced echogenicity. In our patient, levels of both anti-TPO and antithyroglobulin antibodies were significantly elevated, although thyroid function studies were normal, with no suggestive clinical findings of thyroiditis (symptoms of hypothyroidism, painless enlarged thyroid gland), negative thyroid ultrasound, and no family history of any autoimmune disorder. The absence of a positive thyroid ultrasound finding did not require us to further investigate Hashimoto's thyroiditis with fine-needle aspiration cytology.

Euthyroid cases of HE are not uncommon, with one study having reported 19 of 85 cases of HE as euthyroid [7]. However, such patients often have other supportive features, such as seizures, myoclonus, psychosis, goiter, relapsing course, and high CSF protein [7], none of which our patient had. Furthermore, the majority of the patients diagnosed with HE have Hashimoto’s thyroiditis or Graves’ disease [9]. Thus, a history of thyroid symptoms and signs or abnormal thyroid status are not required for the diagnosis of steroid-responsive encephalopathy associated with autoimmune thyroiditis (SREAT) and could raise a possibility of subclinical thyroid disease in the future. Also, the association of Hashimoto's thyroiditis and HE is uncertain as there is insufficient evidence to prove that the thyroid autoantibodies react with the brain tissue [7]. 

Other clinical tests include labs, MRI brain, CSF study, and EEG, which aid in the diagnosis. MRI is normal in approximately 50% of patients with HE [10,11]. However, few studies have reported the presence of a diffuse increased signal on T2-weighted and fluid-attenuated inversion recovery (FLAIR) images in subcortical white matter, generalized cerebral atrophy, and dural enhancement [10-13]. Interestingly, in our patient, MRI showed T1 hyperintensity of bilateral basal ganglia, likely due to mineral deposition. Similar radiological findings have been reported with another case report in the past as well [10]. 

CSF study commonly shows elevated protein levels [8,9]. This is found in 85% of the affected patients [8], however, it was peculiar to note that our patient had a normal study. Our patient also had an EEG finding of generalized slowing with triphasic waves, and this is a common finding in HE, seen in 95% (19/20) of cases [14].

The rapid response to corticosteroid treatment could be due to inflammatory or immunological phenomena and hence HE has been renamed as steroid-responsive encephalopathy associated with autoimmune thyroiditis (SREAT) [14]. Approximately 50% of cases exhibit complete responses to steroid therapy, and 40% achieve complete remission on follow-up [15,16]. However, a recent study has shown that the response to steroids is much lower, with only 31% showing complete clinical response [17]. Thus, while rapid and significant steroid responsiveness points toward a diagnosis of HE, the incomplete response does not rule it out. The same study pointed out that the delay in starting steroids from symptom onset was over a month for three out of five patients who did not improve and less than a month for 13 out of 14 patients who had a partial or complete recovery [17].

To avoid the risk of relapse, the steroid dose has been suggested to be tapered gradually over months or years and monitor for the clinical response. Long-term monotherapy with prednisolone is suggested, but if recurrences occur or side-effects of steroids prevail, combination therapy with immunosuppressive medications, such as azathioprine, cyclophosphamide, and methotrexate can be added [18]. We incorporated a similar idea of tapering the steroid dose gradually over months, and our patient has been maintained on oral prednisolone 10 mg as maintenance. We have not noticed any significant relapse in clinical symptoms during follow-ups for the last three years and no side effects from chronic steroid use.

The amount of increase in the antibody titers from the normal range does not help in identifying the severity of the disease. This possibility ranges from asymptomatic patients having high antibody titers to a severe encephalopathy patient with a mild rise in antibody titers [7]. Hence, the presence of antithyroid antibodies may be linked to another underlying autoimmune predisposition and has been recently termed as nonvasculitic autoimmune meningoencephalitis (NAIM) [19], which requires further speculation.

The most common reversible causes of subacute dementia include depression, normal pressure hydrocephalus, space-occupying lesions, Cushing's disease, metabolic conditions, such as chronic liver failure and chronic kidney disease, infectious causes like tuberculosis, HIV and neurosyphilis, alcohol abuse, and nutritional conditions like vitamin B12 deficiency. We attempt to contribute to the literature by presenting this case of subacute dementia in a euthyroid case with elevated antithyroid antibodies, normal thyroid ultrasound, and normal CSF analysis with marked response to oral steroids.

## Conclusions

Considering the debilitating neurologic presentations, HE appears to be underdiagnosed. Due to a lack of prior history of Hashimoto’s thyroiditis and normal thyroid studies, one should not exclude the possibility of HE, given it is a completely curable condition. However, thyroid status should be monitored regularly for possible subclinical presentation. While the constellation of MRI, EEG, CSF findings can be variable and hence unreliable, response to steroids confirms this diagnosis. Clinicians should be aware of this unlikely presentation of HE with a euthyroid status and include antithyroid antibody studies in their workup for subacute dementia, after excluding other causes. Further studies need to be conducted on developing reliable markers of HE, as elevated antithyroid antibodies could be an incidental finding and steroid responsiveness may not necessarily be seen in all instances.

## References

[1] Olmez I, Moses H, Sriram S, Kirshner H, Lagrange AH, Pawate S (2013). Diagnostic and therapeutic aspects of Hashimoto's encephalopathy. J Neurol Sci.

[2] Brain L, Jellinek EH, Ball K (1966). Hashimoto's disease and encephalopathy. Lancet.

[3] Ferracci F, Bertiato G, Moretto G (2004). Hashimoto's encephalopathy: epidemiologic data and pathogenetic considerations. J Neurol Sci.

[4] Sadan O, Seyman E, Ash EL, Kipervasser S, Neufeld MY (2013). Adult-onset temporal lobe epilepsy, cognitive decline, multi-antiepileptic drug hypersensitivity, and Hashimoto's encephalopathy: two case studies. Epilepsy Behav Case Rep.

[5] Chaudhuri A, Behan PO (2003). The clinical spectrum, diagnosis, pathogenesis and treatment of Hashimoto's encephalopathy (recurrent acute disseminated encephalomyelitis). Curr Med Chem.

[6] Kothbauer-Margreiter I, Sturzenegger M, Komor J, Baumgartner R, Hess CW (1996). Encephalopathy associated with Hashimoto thyroiditis: diagnosis and treatment. J Neurol.

[7] Chong JY, Rowland LP, Utiger RD (2003). Hashimoto encephalopathy: syndrome or myth?. Arch Neurol.

[8] Lee SW, Donlon S, Caplan JP (2011). Steroid responsive encephalopathy associated with autoimmune thyroiditis (SREAT) or Hashimoto's encephalopathy: a case and review. Psychosomatics.

[9] Zhou JY, Xu B, Lopes J, Blamoun J, Li L (2017). Hashimoto encephalopathy: literature review. Acta Neurol Scand.

[10] Ramalho J, Castillo M (2011). Hashimoto's encephalopathy. Radiol Case Rep.

[11] Tamagno G, Celik Y, Simó R (2010). Encephalopathy associated with autoimmune thyroid disease in patients with Graves' disease: clinical manifestations, follow-up, and outcomes. BMC Neurol.

[12] Chen N, Qin W, Wei C, Wang X, Li K (2011). Time course of Hashimoto's encephalopathy revealed by MRI: report of two cases. J Neurol Sci.

[13] Matsunaga A, Ikawa M, Kawamura Y (2019). Serial brain MRI changes related to autoimmune pathophysiology in Hashimoto encephalopathy with anti-NAE antibodies: a case-series study. J Neurol Sci.

[14] Castillo P, Woodruff B, Caselli R (2006). Steroid-responsive encephalopathy associated with autoimmune thyroiditis. Arch Neurol.

[15] Berger I, Castiel Y, Dor T (2010). Paediatric Hashimoto encephalopathy, refractory epilepsy and immunoglobulin treatment-unusual case report and review of the literature. Acta Paediatr.

[16] Vernino S, Geschwind M, Boeve B (2007). Autoimmune encephalopathies. Neurologist.

[17] Mattozzi S, Sabater L, Escudero D (2020). Hashimoto encephalopathy in the 21st century. Neurology.

[18] Marshall GA, Doyle JJ (2006). Long-term treatment of Hashimoto's encephalopathy. J Neuropsychiatry Clin Neurosci.

[19] Chong JY, Rowland LP (2006). What's in a NAIM? Hashimoto encephalopathy, steroid-responsive encephalopathy associated with autoimmune thyroiditis, or nonvasculitic autoimmune meningoencephalitis?. Arch Neurol.

